# Age estimation of captive Asian elephants (*Elephas maximus*) based on DNA methylation: An exploratory analysis using methylation-sensitive high-resolution melting (MS-HRM)

**DOI:** 10.1371/journal.pone.0294994

**Published:** 2023-12-11

**Authors:** Kana Arai, Huiyuan Qi, Miho Inoue-Murayama

**Affiliations:** Wildlife Research Center, Kyoto University, Kyoto, Japan; University of Veterinary Medicine Vienna: Veterinarmedizinische Universitat Wien, AUSTRIA

## Abstract

Age is an important parameter for bettering the understanding of biodemographic trends—development, survival, reproduction and environmental effects—critical for conservation. However, current age estimation methods are challenging to apply to many species, and no standardised technique has been adopted yet. This study examined the potential use of methylation-sensitive high-resolution melting (MS-HRM), a labour-, time-, and cost-effective method to estimate chronological age from DNA methylation in Asian elephants (*Elephas maximus*). The objective of this study was to investigate the accuracy and validation of MS-HRM use for age determination in long-lived species, such as Asian elephants. The average lifespan of Asian elephants is between 50–70 years but some have been known to survive for more than 80 years. DNA was extracted from 53 blood samples of captive Asian elephants across 11 zoos in Japan, with known ages ranging from a few months to 65 years. Methylation rates of two candidate age-related epigenetic genes, *RALYL* and *TET2*, were significantly correlated with chronological age. Finally, we established a linear, unisex age estimation model with a mean absolute error (MAE) of 7.36 years. This exploratory study suggests an avenue to further explore MS-HRM as an alternative method to estimate the chronological age of Asian elephants.

## Introduction

Asian elephant (*Elephas maximus*) populations are under considerable threat due to poaching, habitat loss, fragmentation, and human-elephant conflict. As a result, they have been listed as endangered species on the International Union for Conservation of Nature (IUCN) Red List since 1986, and their population has declined by at least 50% over the last 75 years [1]. Accurate population density and age estimations are required to supplement ongoing conservation measures and protect the existing populations. Reliable chronological age estimation is essential in population recovery planning to assess the impacts of poaching, habitat fragmentation, and captive breeding management [2]. Therefore, determining chronological age is an important ecological tool for conservation and management. Moreover, knowing individuals’ chronological age is useful to understand ecological traits as many biological characteristics, such as size, behaviour, and sexual maturity are age-related [3]. These characteristics could impact an individual’s ability to survive [4] and reproduce effectively [5]. Consequently, the population is affected; therefore, accurately determining age could assist in estimating the population age structure, which can further infer past events and present and future extinction risks [6,7].

Historically, most chronological age estimation methods required either long-term visual surveys, mark-recapture strategies, or examination of dead remains [8–11]. Most of these methods require investigating morphological characteristics and tracking individuals. However, this is often time-consuming, and a limited number of species exhibit observable and significant age-related changes. Currently, the main method to estimate chronological age in Asian elephants is visually estimated by observing size characteristics, such as shoulder height [9,12]. However, this method is prone to gross overestimation, especially in long-lived species such as Asian elephants, as they mature slowly, and the visual determination of mature adults becomes difficult and subjective [9]. Cranio-dental-based age criteria for Asian elephants have been developed; however, they can only be used on dead specimens, limiting their application [13,14]. Other chronological age estimation techniques developed for Asian elephants include eye lens [15], tusk size, ear pigmentation [9], and dung bolus circumference [16,17]. However, most of these methods can either be highly subjective, inaccurate, or difficult to perform in the field. Developing a fast, objective, accurate, and cost-effective method to estimate the chronological age of Asian elephants is needed.

Various molecular ageing markers, such as mutation accumulation in mitochondrial or nuclear DNA, changes in mitochondrial DNA copy number, telomere length, and DNA methylation, have been assessed for age estimation [6,18,19]. The DNA methylation-derived epigenetic clock presents a highly promising molecular biomarker of ageing, especially in live animals [20,21]. DNA methylation is a well-studied epigenetic modification that involves the transfer of a methyl group onto cytosine to form 5-methylcytosine, affecting the activity of a DNA segment without changing the actual sequence [22] and plays a significant role in various biological processes, such as gene expression regulation, genomic imprinting, X-chromosome inactivation, and ageing [23,24]. Changes in ageing-associated DNA methylation levels occur at CpG sites (where a cytosine is next to a guanine in the DNA sequence). Clusters of these sites are called CpG islands and are often observed in the promoter of the gene [24] and determine the level and integrity of gene expression [18,25] in response to developmental and environmental signals [26]. Differences in DNA methylation levels at specific CpG islands in certain genes and ages, mainly in mammals, are well reported. Outstudies have been focused on species, such as dogs (*Canis familiaris*) [27], cats (*Felis catus*) [28], chimpanzees (*Pan troglodytes*) [29], bats (*Myotis bechsteinii*) [30,31], seabirds (*Ardenna tenuirostris*) [32], green turtles (*Chelonia mydas*) [33], fish [34,35], and several cetacean species [36–39].

DNA methylation profiling techniques have evolved throughout the years. Increasing studies using molecular techniques such as pyrosequencing, DNA microarray chips, reduced-representation bisulphite sequencing (RRBS), and whole-genome bisulphite sequencing (WGBS) have accumulated knowledge on the relationship between methylation patterns and chronological age. A single gene technique to measure DNA methylation using pyrosequencing was one of the first studies to measure chronological age in non-model species [37]. Methylation arrays used to be limited to humans [18] but have now spread its applicability towards mammalian species using a specific methylation mammal array, making it one of the preferred techniques to this day [40]. Recently, developments in next-generation sequencing (NGS) based DNA methylation analysis using RRBS or WGBS techniques have allowed rapid and efficient generation of massive amounts of high-quality sequencing data. However, most of these methods require specialised instruments, analysis machines, and complex computational analysis, bioinformatics, and evaluation for age estimation. Moreover, high costs (pyrosequencing: approximately 3–4.5 h per run and $14 per sample based on two markers; DNA microarray chips: approximately, 1 h per chip and $150 per sample; RRBS: 4 h per run and $300–600 per sample; WGBS: $31.70 per GB, assuming 100 GB of raw data is obtained) have prevented these methods from being routinely used. Methylation-sensitive high-resolution melting (MS-HRM) is a real-time polymerase chain reaction (PCR) based method that detects methylation levels easily, quickly, and cost-effectively (approximately 2 h per run at $7 per sample based on two markers), making it a promising technique for age estimation [41,42]. MS-HRM and pyrosequencing are some of the most convenient methods with comparable accuracy [43] but MS-HRM has better advantages in time and cost-effectiveness. Furthermore, using MS-HRM, standard techniques, specialised skills, costly equipment, and complicated bioinformatics are not required. During MS-HRM, the bisulphite-treated DNA is amplified using PCR, followed by melting analysis, where the unmethylated cytosines are converted into uracil during bisulphite conversion. In contrast, the methylated cytosines remain unchanged [44,45]. This allows for different base compositions and information on methylation status directly related to the sequence, enabling a quantitative methylation assessment [41,46]. The unique characteristic of MS-HRM that separates it from other DNA methylation techniques is that it measures the overall methylation status of amplified PCR products rather than the individual CpG markers. This allows the information, regarding many CpG markers observed in the genetic region of interest to be integrated and analysed with a pair of PCR primers in a single measurement [41].

DNA methylation studies on Asian elephants are limited. To our knowledge, only a single study used an array-based technology that permits the quantification of methylation levels at specific CpG sites [47]. Methylation array-based technology remains widely used across many mammalian species as an accurate method to measure DNA methylation [40]. However, as mentioned above, it may not be the most time- and cost-effective approach, more so when applications to conservation and management strategies, including for existing wild populations are intended. Although age estimator techniques studied in Asian elephants, such as microarrays or long-term observations also provide valuable estimates, it may not be feasible for all populations. Due to its advantages, a traditional polymerase reaction, a universal and cost- and time- effective method, such as MS-HRM may be the key technique for age estimation in real-life situations for both captive and wildlife investigation.

To our knowledge, the use of MS-HRM to determine chronological age has only been successfully attempted on humans [41,46], cats and snow leopards (*Panthera uncia*) [28]. An age estimation model for Asian elephants using MS-HRM would be valuable for management strategies. The main purpose of this study was to investigate the potential use of MS-HRM as an alternative to the current age estimation methods. As there are only a few studies on DNA methylation in Asian elephants, the objectives of this study were designed to test the DNA methylation-based age estimation technique using MS-HRM. We investigated the relationship between epigenetic modifications in age-related genes and chronological age, and its potential as an age estimation model in Asian elephants. The methylation rate of two candidate age-related epigenetic markers, *RALYL* and *TET2*, was determined to assess the following: (1) the correlation between chronological age and methylation levels; (2) the accuracy of MS-HRM for chronological age estimation in Asian elephants; and (3) how MS-HRM use in elephants compares to its use in other species for age determination. Our study findings will assist in considering using MS-HRM as a potential alternative approach to estimating chronological age in Asian elephants.

## Materials and methods

**Fig 1:**
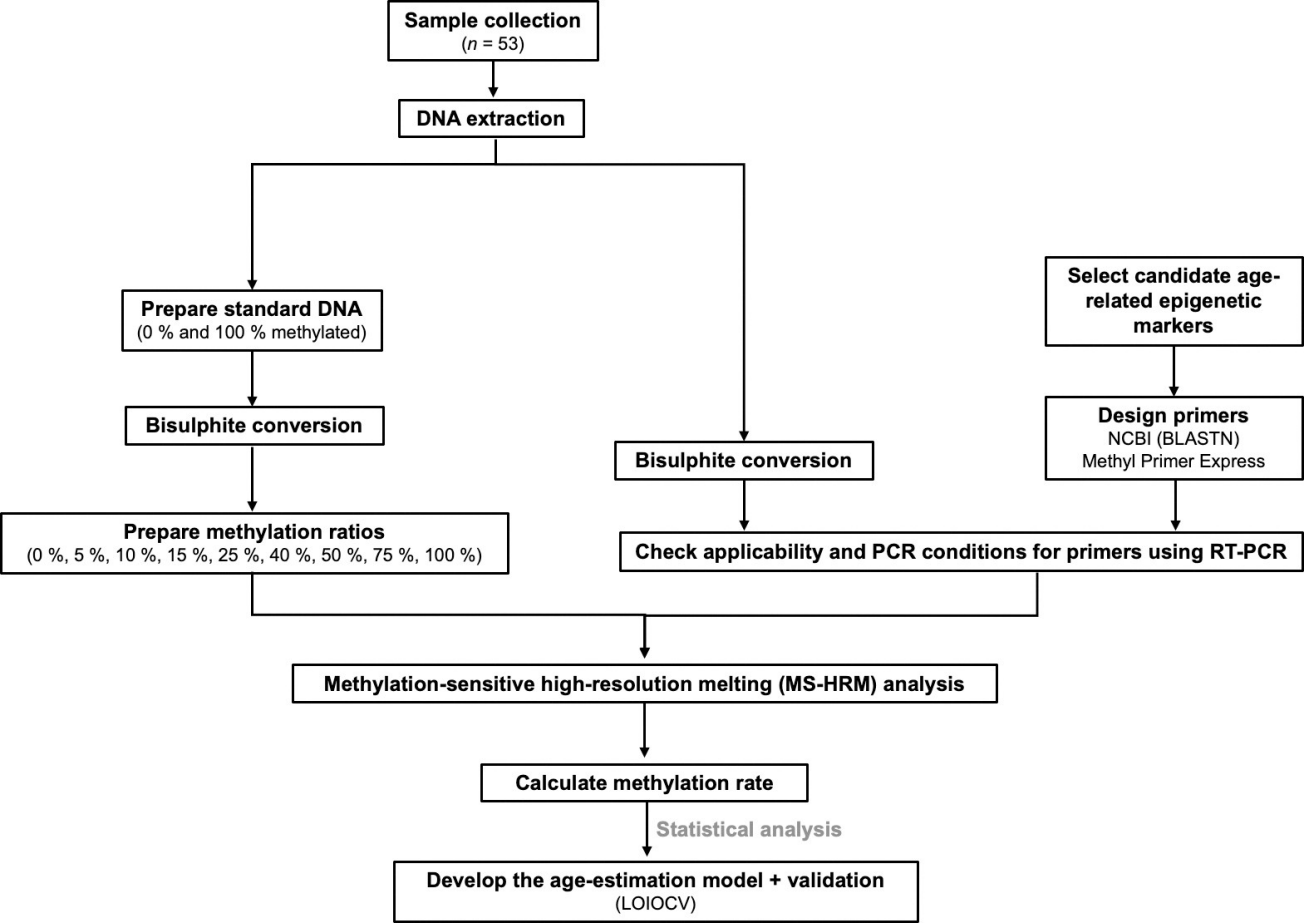
Graphical representation of the methodology. Workflow to build an age estimation model using methylation-sensitive high-resolution melting (MS-HRM).

A graphical representation of the workflow is represented in Fig 1, which summarises the steps required to construct an age estimation model using MS-HRM.

### Ethics statement

All methods were performed following relevant guidelines and regulations and reported under ARRIVE guidelines. All experimental protocols and sample collections were approved by the ethical committee and conducted in strict accordance with the guidelines for the ethics of animal research established by the Wildlife Research Center of Kyoto University (approval number WRC-2022-010A). The samples collected from Asian elephants were collected and used with the agreement of each zoo.

### Sample collection and DNA extraction

Blood samples (*n* = 53) were collected from 2004–2022 across 11 zoos in Japan from 25 captive Asian elephants (S1 Table) during regular veterinary health examinations. As of 2021, there are only 31 zoos in Japan that house 81 captive Asian elephant individuals [48]. Most of the zoos in Japan undergo husbandry training to collect blood samples during health examinations from captive Asian elephants to minimise invasive sampling. However, not all elephants are trained, and even well-trained individuals suddenly resist getting their blood drawn. Thus, to minimise such invasive sampling, our sample collection focused on 25 well-trained individuals who were not reluctant to collect blood at the time. Of the 25 individuals, three were male and 22 were female. As blood samples from individuals were collected multiple times over the years, samples ranging from a few months to 65 years of age were provided by zoos. Known birthdates or estimated birth years were informed by zoo staff taken from those recorded in the studbooks. Individuals had known birthdates if they were born in a captive facility in Japan or a range country (usually from Myanmar, Laos, or Thailand), while those recorded with an estimated birth year were wild-born imported individuals. Age for estimated birth years was most likely done by the broker at the time of importation or by zoo staff on arrival, based on individual morphometric measurements. Our sample set had a larger proportion of known birthdates than estimated birth years (six estimated individuals and 19 known individuals). Individuals with estimated birth years were born before the late 1970s. This reflects the management history of the population as the implementation of captive breeding as a population management tool was not fully implemented until the 1980s.

All samples were stored in a −80°C or −20°C freezer until use. DNA from the collected blood samples was extracted using the DNeasy Blood & Tissue Kit (QIAGEN, GmbH, Hilden, Germany) according to the manufacturer’s protocol. The DNA concentration was determined using a Qubit 4 Fluorometer with the Qubit dsDNA Assay Kit (Thermo Fisher Scientific, San Jose, CA, USA) using 1 μL of input DNA and was stored at −20°C until further use.

### Standard DNA

A standard DNA was prepared to calculate the methylation status of samples from MS-HRM [28,41]. The negative (fully unmethylated) control standard DNA was synthesised using a whole genome amplification treatment on one DNA sample using the REPLI-g Mini Kit (QIAGEN, GmbH, Hilden, Germany). The positive (fully methylated) control standard DNA was obtained by fully methylating the above-synthesised DNA sample with CpG methyltransferase (*M*.*Sssl*, New England Biolabs, Beverly, MA, USA). The obtained standard DNA (0% and 100% methylated) was then purified using a High Pure PCR Product Purification Kit (Roche Molecular Systems, Pleasanton, CA, USA).

### Bisulphite conversion

All blood DNA samples, and purified standard DNA (0% and 100%) were bisulphite-converted using the EZ DNA Methylation-Gold Kit (Zymo Research, Irvine, CA, USA) according to the manufacturer’s protocol. The DNA input used to prepare bisulphite-converted DNA samples was 300 ng. In comparison, a higher DNA input of 500 ng was used to prepare bisulphite-converted standard control DNA, considering that methylation ratios were needed to be prepared from 0% and 100% bisulphite-converted standard control DNA. The concentration of bisulphite-converted DNA was then measured with a Qubit 4 Fluorometer using the Qubit ssDNA Assay Kit (Thermo Fisher Scientific, San Jose, CA, USA) and subsequently adjusted to 5 ng/μL.

### Identification of age-related epigenetic markers and primer designs

Genes with age-related epigenetic changes were identified via a literature search using databases such as Google Scholar and PubMed. Search terms included ‘age estimation’ or ‘age determination’ and ‘DNA methylation’ or ‘epigenetics’, and further focused on literature related to ‘mammals’, ‘MS-HRM’, and ‘pyrosequencing’ so primers can easily be modified for amplification of bisulphite-converted DNA. The study on elephant epigenetics using microarrays [47] was included in the search for comparative purposes. We selected target genomic locations adjacent to two genes namely *RALYL* (RALY RNA-binding protein-like) and *TET2* (Tet methylcytosine dioxygenase 2). The methylation levels of CpG sites adjacent to these genes change with age. *RALYL* has previously been used to estimate the age of domestic dogs [49], cats, and snow leopards [28], and it is hypermethylated in cancer tissues [50]. *TET2* has been studied in cetacean species [36,37,39], bats [30], and for its role in cancer development, where *TET2* mutations were associated with cancer tissue hypermethylation [51,52], which leads to epigenetic age acceleration in humans [53]. We identified homologous sequences against the genomic regions of Asian elephants (GCA_014332765.1) [54] containing the target CpG sites using the Standard Nucleotide Basic Local Alignment Search Tool (BLASTN) provided by the National Center for Biotechnology Information (NCBI) [55]. PCR primers for amplifying Asian elephant sequences were designed using Methyl Primer Express v1.0 (Thermo Fisher Scientific, San Jose, CA, USA) and/or manually when required (Table 1).

**Table 1:** Primer information and polymerase chain reaction (PCR) conditions.

Gene	Primers^a^	Length	n(CpGs)	PCR conditions	NCBI sequence ID: position	References
*RALYL*	F: gCgatggttTtgtagaTaagg R: cgttttttccataaaaccaAttA	109	8	95°C (5min), [95°C (10 s); 50°C (30 s); 72°C (10 s)] * 45 cycles	NC_064833.1: 57493402–57493509	Lowe et al., (2018); Qi et al., (2021)
*TET2*	F: gtggagaggtTaTtgagaagaaagt R: aaaataaggagggcttctgc	202	6	95°C (5min), [95°C (10 s); 60°C (30 s); 72°C (10 s)] * 50 cycles	NC_064833.1: 46696650–46696870 (reverse complement)	Polanowski et al., (2014); Wright et al., (2018)

^a^Capital letter: Bisulphite-converted letter (F: C → T, R: G → A). NCBI, National Center for Biotechnology Information.

Other candidate genes were selected during the literature search. However, amplification and dissociation curves for these genes were unsatisfactory following real-time PCR (RT-PCR) (Thermal Cycler Dice Real Time System TP800, Takara Bio Inc, Kusatsu, Shiga, Japan), and they were disregarded. Results remained unsatisfactory even when PCR conditions were modified several times (S1 Table in S1 File). Even when RT-PCR results were satisfactory, MS-HRM results would be negative or show no correlation between methylation and chronological age (S1 Fig in S2 File).

### Methylation-sensitive high-resolution melting (MS-HRM)

To evaluate the applicability and PCR conditions for *RALYL* and *TET2*, RT-PCR was performed using the Thermal Cycler Dice System TP800 (Takara Bio Inc, Kusatsu, Shiga, Japan) before using MS-HRM. The amplification plots and dissociation curves were observed to confirm that the PCR conditions amplified the target region. The PCR conditions are listed in Table 1. In MS-HRM, bisulphite-converted DNA samples were PCR-amplified, followed by melting analysis. This follows the methods of Hamano et al. [41] and Qi et al. [28], for which procedures were modified accordingly (Table 1). PCR amplification was carried out using a Roche LightCycler 480 II (Roche Molecular Systems, Branchburg, NJ, USA) equipped with Gene Scanning Software (version 1.5.1.62 SP2) in a 25 μL total volume containing 1xEpiTect HRM PCR Master Mix (EpiTect HRM PCR kit; QIAGEN GmbH, Hilden, Germany), 750 nM of each primer, and 2 μL of template DNA (5 ng/μL of bisulphite-converted DNA). In addition to the samples, standard curves with known methylation ratios (0%, 5%, 10%, 15%, 25%, 40%, 50%, 75%, and 100%) were included in the assay plates and were later used to calculate the methylation rate of each *RALYL* and *TET2* genes per sample. The ratios were prepared by mixing 0% and 100% methylated standard DNA in appropriate ratios to create a standard series. All reactions were performed in duplicate.

The chosen detection format was set to SYBR Green I/HRM dye. Following the initial PCR activation step at 95°C for 5 min to activate the polymerase. The 3-step cycling PCR conditions used were the same as those listed in Table 1. Following PCR amplification, HRM analysis was initiated by cooling the samples to 65°C for 1 s and then heated to 95°C at a ramp rate of 0.02°C/s. The continuous fluorescence at this step allows for data acquisition, where the fluorescence intensity was measured at 25 acquisitions per second during the entire process. When HRM analysis was performed, Gene Scanning Software first normalised raw melt curves to compare samples. In this normalising process, after MS-HRM was performed, the pre- and post-melt temperature regions were set to 67–68°C and 82–83°C for *RALYL*, respectively. For *TET2*, these were set to 66–67°C and 80–81°C, respectively. The threshold was set to zero for both genes, as the shape of the melting curve was important for the analysis of the overall methylation status of the amplicon. If the threshold was not set to zero and the temperature shift process was not performed, the shape of the melting curve would be distorted [41]. A difference curve was then derived from the first derivative of the melting curves after setting the data to the 0% methylated standard sample as a baseline. The maximum absolute value of the relative signal difference from the difference curves was defined as the ‘Df value’ for each sample.

### Methylation rate calculation

PCR bias often occurs when amplifying bisulphite-treated DNA, as unmethylated DNA tends to amplify more efficiently than in methylated DNA [56,57]. A standard curve for each target site should be obtained to analyse methylation profiles accurately before measuring unknown methylated samples with MS-HRM [41]. From the MS-HRM analysis, standard curves were prepared for each experiment to calculate the methylation status of samples using Eq 1.

The Df values of the standard series were plotted for both *RALYL* and *TET2*, and a non-linear regression model developed by Warnecke et al. [56] (modified by Hamano et al. [41]) was used as follows:

$$\frac{a*M}{100-M}=\frac{Df}{Dfmax-Df}$$ (1)

where ‘M’ represents the methylation rate (percentage DNA methylation of each region of interest), ‘Dfmax’ is the Df value of the 100% methylated standard sample, and ‘a’ is the coefficient. Conducting the regression model and calculating the estimated value ‘a’ were performed using the ‘nls’ command in R version 4.2.0 [58]. The methylation rates (M) of the samples for *RALYL* and *TET2* were calculated by substituting the Df value and ‘a’ value into the equation above.

### Correlation between methylation rate and chronological age

For each target region, the correlation between methylation rate and chronological age was analysed using Pearson’s product-moment correlation coefficients by considering the *p*-value (*p* <0.05 was considered significant) and *cor* value. Figures were created using the package ggplot2 [59].

### Age estimation model development and validation

To construct the age estimation model, a support vector regression (SVR) was built using the ‘e1071’ package [60]. SVR is suggested to be a robust choice that has high estimation accuracy with low-level overfit [61]. The SVR model parameters were optimised using the ‘tune.svm’ command with the optimisable parameters ‘cost’ and ‘epsilon’, and the fixed setting ‘type = eps-regression, kernel = radial, gamma = 0.5 or 1’. The optimised parameters are summarised in the S2 Table in S1 File. As some individuals were sampled more than once, cross-validation of the SVR model was performed using leave-one-individual-out cross-validation (LOIOCV). When the dataset used for model building and evaluation is small, the LOIOCV approach is recommended for model evaluation as it maximises the size of the training set in each iteration and further tests the robustness of the model to validate the overfitting of the optimised model. Moreover, LOIOCV takes into account individuals sampled multiple times and ensures that an individual is used as a training set while all other individuals are used as the testing set. This is repeated as many times as the number of individuals. To evaluate the deviation source in the model, a linear regression model with the age-estimation difference (predicted age–chronological age) as the response variable was constructed using the ‘lm’ command, and model selection was conducted using the ‘MuMIn’ package. The prediction accuracy of the regression model was assessed using the coefficient of determination (*R*^2^) and the mean absolute error (MAE) after LOIOCV analysis. Each age-responsive gene using chronological age was used as the independent known variable, while the predicted age for each region at different age-responsive genes was the dependent variable. To compare the relative predictive accuracy of each data type, the *R*^2^ and *p*-value between predicted and chronological age were compared using simple linear regressions for each of the genetic regions and when both regions were combined into the model.

To evaluate whether sex influences the deviation of the age estimation model, generalised linear regressions were generated. Here, we denoted ‘Δage residual’ as the residual resulting from a linear model when regressing predicted age on chronological age, after adjusting chronological age as a covariate. The practical utility of the model was then further assessed by classifying individuals into appropriate age classes: calf (<1 year), juvenile (1–5 years), subadult (5–15 years), and adult (>15 years) based on Sukumar [62] as it could provide valuable information on population viability [63,64]. The predictive power of achieving the correct age classification was assessed by calculating the kappa values using the ‘vcd’ package, where values closer to 1 indicate good predictive power [65,66]. To increase the confidence, a one-way analysis of variance (ANOVA) was used, which was then further analysed using a post-hoc test with the ‘TukeyHSD’ command and ‘multcompView’ package.

### Assessment of within-individual change: Methylation-based longitudinal age prediction

As blood samples of certain individuals were collected over time (S1 Table), individuals with at least two samples were chosen to further validate the model’s predictive accuracy by assessing the methylation-based longitudinal age prediction. A binomial test was conducted using the ‘binom.test’ command to evaluate if the age estimation model would correctly predict that samples collected from an individual at a later date were indeed from an older sample. A simple regression analysis was performed to evaluate if within-individual change comparisons would affect the overall model (the relationship between predicted age and chronological age).

## Results

### Standard curves

**Fig 2:**
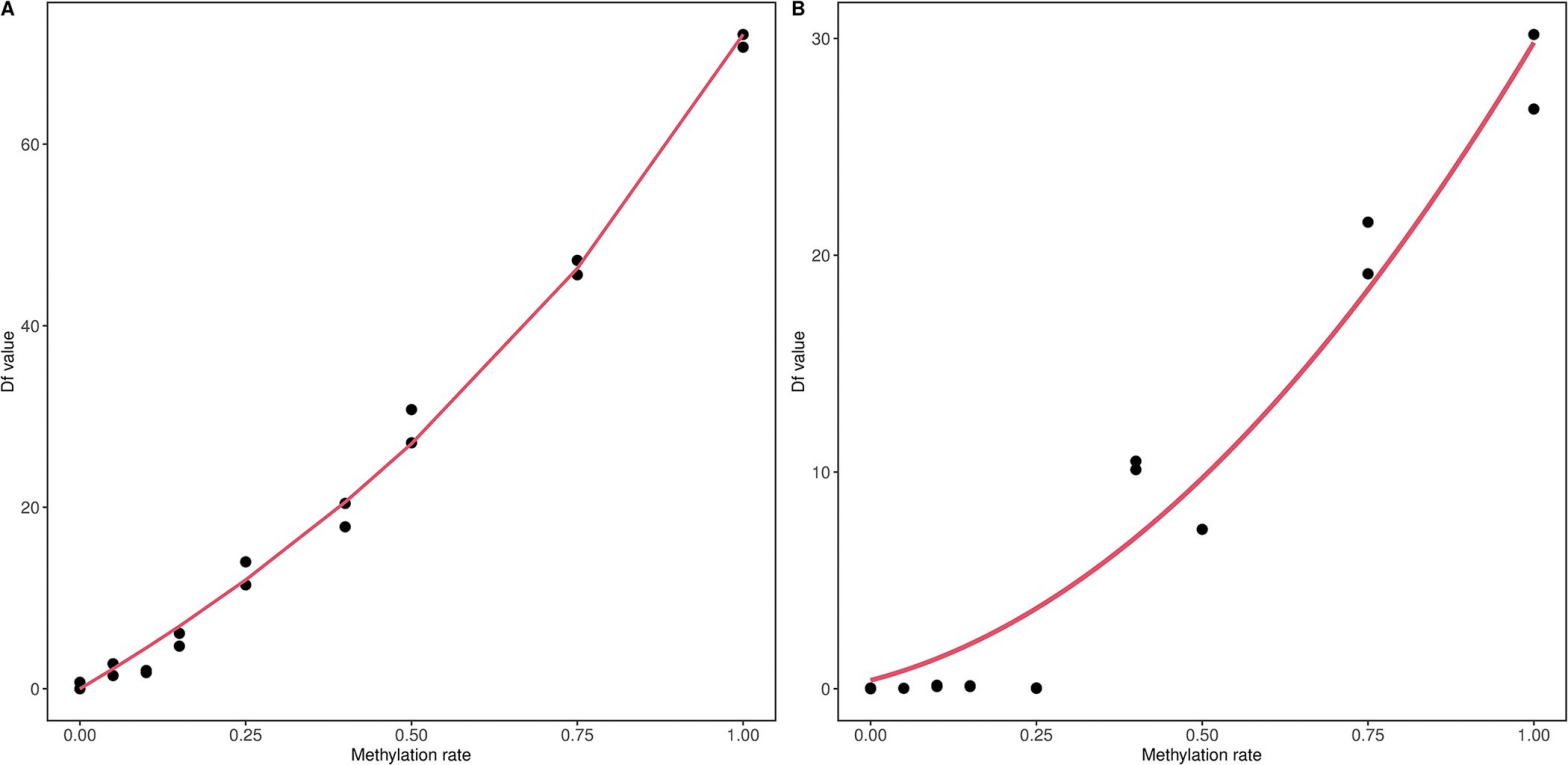
Standard curves of *RALYL* and *TET2*. The standard curves describe the relationship between methylation rate and Df value for (A) *RALYL* and (B) *TET2*. The Df value represents the raw fluorescence data from methylation-sensitive high-resolution melting (MS-HRM).

For MS-HRM analysis, a standard curve was prepared for each genetic region to calculate the methylation status of the samples, according to Eq (1) (refer to ‘Methylation rate calculation’ in the ‘Materials and methods’ section). The standard curves of *RALYL* and *TET2* for Asian elephants are shown in Fig 2. The standard line of *TET2* was less linear compared to that in *RALYL*, likely due to PCR bias. Both genetic regions could clearly detect methylation rate differences between 40% and 100% by Df values. In comparison, those under 10% were hard to detect. *TET2* showed a higher PCR bias and did not fit the regression line well (Fig 2). However, as most of the samples from *TET2* were distributed between 10% and 35%, it appeared to yield predictions close to the standard curve. Standard curves influenced by PCR bias were seen in a previous study with other genetic regions [41]. The estimated ‘a’ value using Eq (1) for *RALYL* and *TET2* is summarised in the S3 Table in S1 File.

### Correlation between methylation rate and age

**Fig 3:**
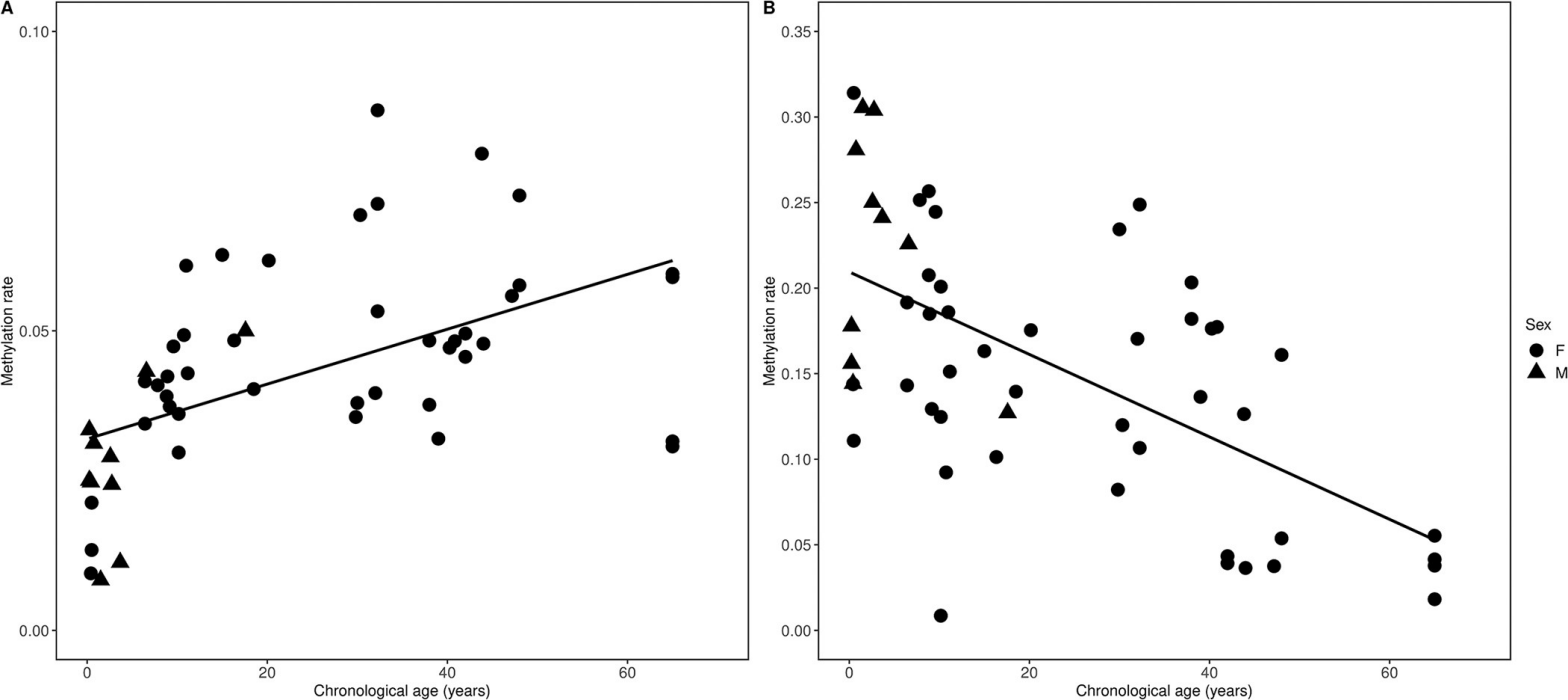
Correlation between methylation rate and chronological age. Correlation between methylation rate and chronological age for (A) *RALYL* (*cor* = 0.52, *p* <0.001) and (B) *TET2* (*cor* = −0.60, *p* <0.001) in Asian elephants.

The methylation rates of *RALYL* (*cor* = 0.52, *p* <0.001) and *TET2* (*cor* = −0.60, *p* <0.001) in Asian elephants were significantly correlated with chronological age (Fig 3). *RALYL* sites revealed that increasing age was associated with increased methylation, whereas *TET2* sites suggested that increasing age was associated with decreased methylation.

### Age estimation model

The age estimation model was constructed based on the SVR model and performances were compared after LOIOCV analysis (Table 2 and S4 Table in S1 File). The model with both genetic regions was the best-performing model and was chosen as the final age estimation model.

**Table 2:** Summary and comparison of model output. *R*^2^ and mean absolute error (MAE) values for each age estimation model after leave-one-individual-out cross-validation (LOIOCV).

Model	*R* ^2^	MAE
*RALYL*	0.22	12.39
*TET2*	0.24	8.92
Final age estimation model (*RALYL* + *TET2*)	0.74	7.36

**Fig 4:**
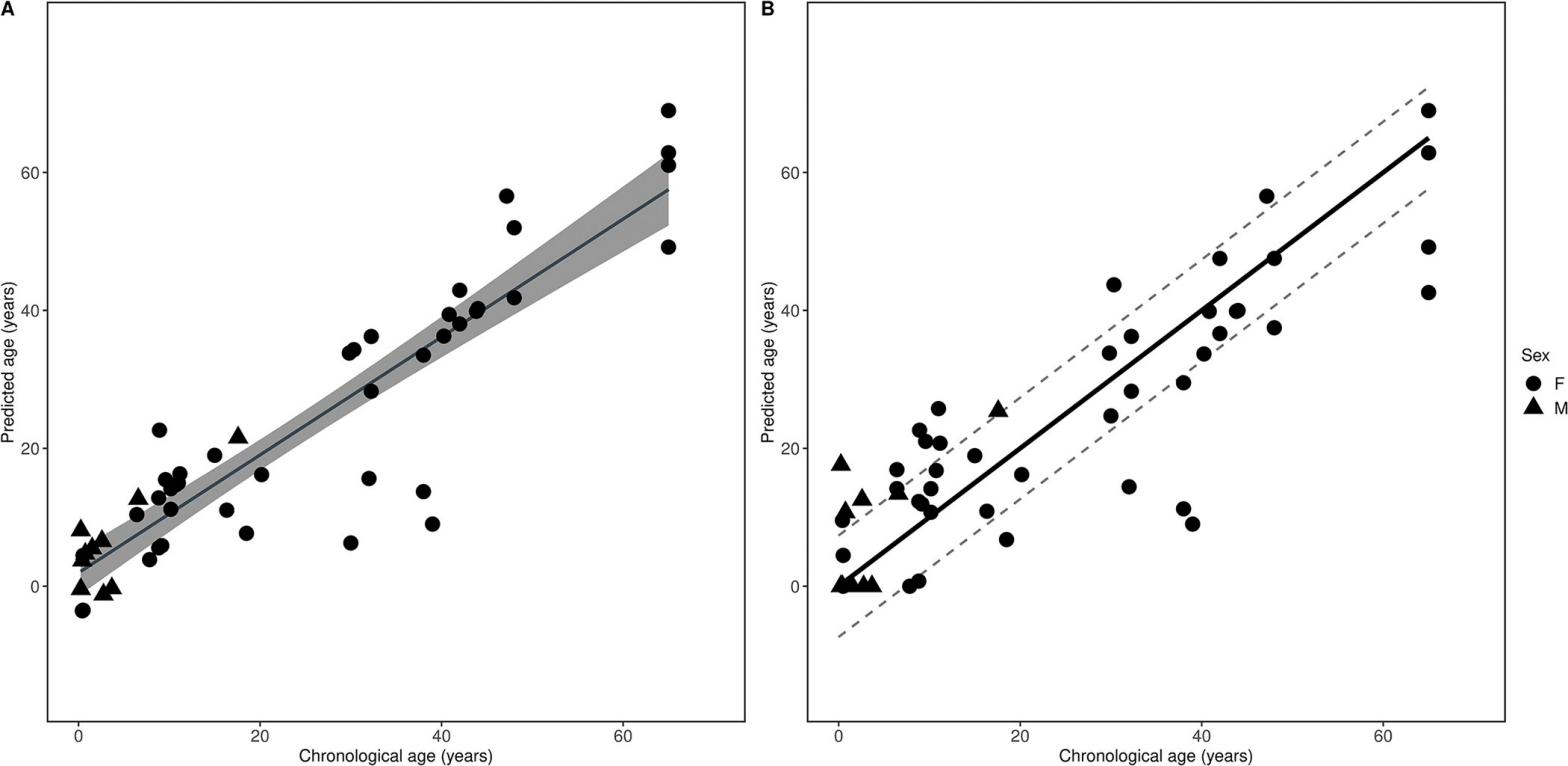
Final age estimation model. Analysis of estimation accuracy and model performance on the final age estimation model combining *RALYL* and *TET2*. (A) SVR model before leave-one-individual-out cross-validation (LOIOCV) analysis (*R*^2^ = 0.82, *p* <0.001). 95% confidence limits are shown on the regression line (grey). (B) Results after LOIOCV analysis. The black line represents a y = x diagonal line, and the region between the grey dash lines represents the mean absolute error (MAE) range, which was 7.36 years (*R*^2^ = 0.74, *p* <0.001).

As the methylation rate of both genetic regions was correlated with age, both regions were used as explanatory variables in the final age estimation model. The accuracy of the model for combining *RALYL* and *TET2* was assessed using the equation generated from the regression analysis with all samples to generate the SVR model (Fig 4A). The regression of *R*^2^ = 0.82 (*p* <0.001) indicated that most of the response can be attributed to age. The overall estimation accuracy before LOIOCV was 5.03 years by calculating the MAE (Fig 4A). The overall estimation accuracy of the final age estimation model after LOIOCV was estimated at 7.36 years (*R*^2^ = 0.74, *p* <0.001) (Fig 4B and Table 2).

### Influence of sex on the age estimation model

**Fig 5:**
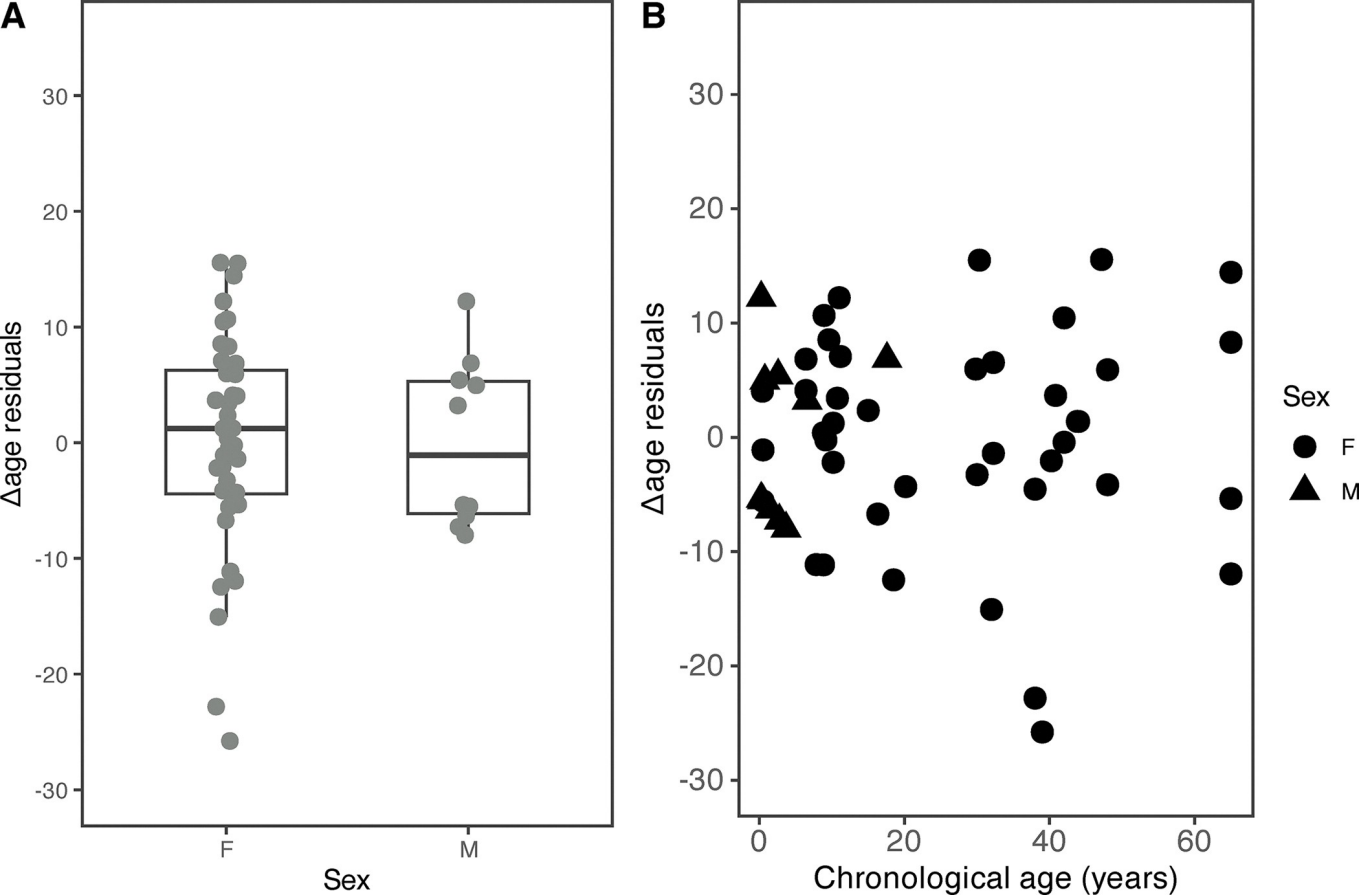
Influence of sex on the final age estimation model. Δage residuals, defined as the residual from regressing predicted age on chronological age was calculated. Chronological age was adjusted as a covariate. (A) Compares the distribution of Δage residuals between females and males. The boxplots show group medians (solid line), inter-quartile range (box outline) and spread of data with outliers (whiskers) for each group. (B) The relationship between Δage residuals and chronological age. Linear regression analysis indicated that sex did not affect Δage residuals significantly (*p* = 0.99).

Linear regression analyses were used to identify the factors that affect Δage residuals in the final age estimation model. There was no significant influence of sex on the Δage residuals thus, not affecting the model (*p* = 0.99) (Fig 5 and S5 Table in S1 File).

### Further age estimation model validation

**Fig 6:**
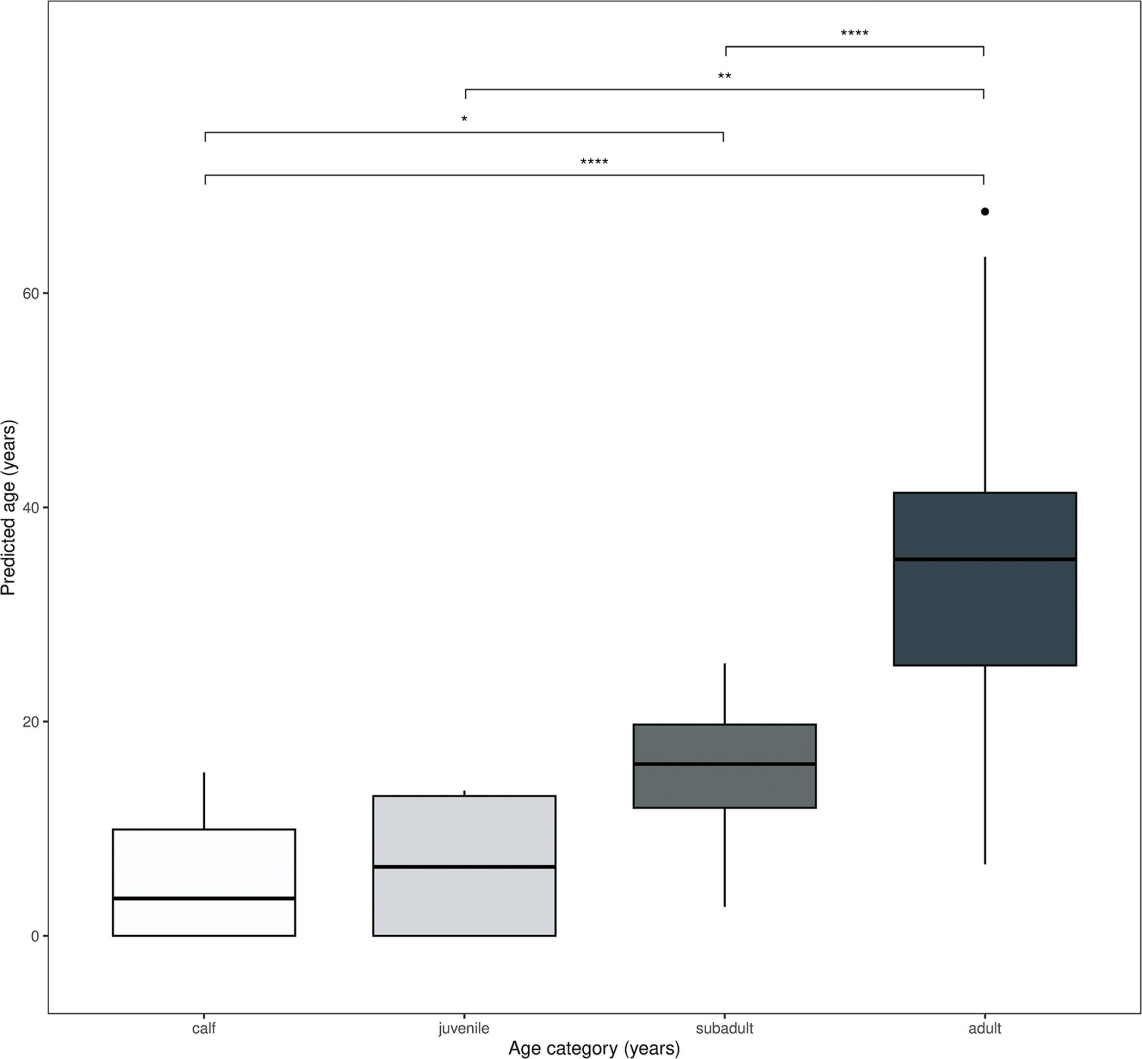
Age group validation of the final age estimation model. Box plots represent the predicted age of captive Asian elephants categorised into four categories of known age: calf (<1 year), juvenile (1–5 years), subadult (5–15 years), and adult (>15 years). The boxplots show group medians (solid line), inter-quartile range (box outline) and spread of data with outliers (whiskers) for each group. The final model developed by combining both genes, *RALYL* and *TET2*, could differentiate age classes according to the one-way ANOVA (*F* = 25.14, *p* <0.001) with a good level of prediction (kappa value = 0.620, *p* <0.001). A kappa value closer to 1 indicates good predictive power. Further analysis with the Tukey-Kramer post-hoc test indicates a statistically significant difference between calf-subadult (*p <0.05), calf-adult (****p <0.0001), juvenile-adult (**p < 0.01), and subadult-adult (****p <0.0001).

The final model tended to slightly overestimate or underestimate the age of individuals. However, when the data was split into appropriate age classes of calf, juvenile, subadult, and adult, the final age estimation model developed from both genes showed that it could differentiate certain age classes (one-way ANOVA: *F* = 16.02, *p* <0.001) with a kappa value of 0.620 (0.510–0.731, *p* <0.001) (Fig 6), which indicates a good level of prediction for practical purposes. However, there were still difficulties in differentiating between the younger age categories, such as calf-juvenile and juvenile-subadult (Fig 6).

### Assessment of within-individual change: Methylation-based longitudinal age prediction

**Fig 7:**
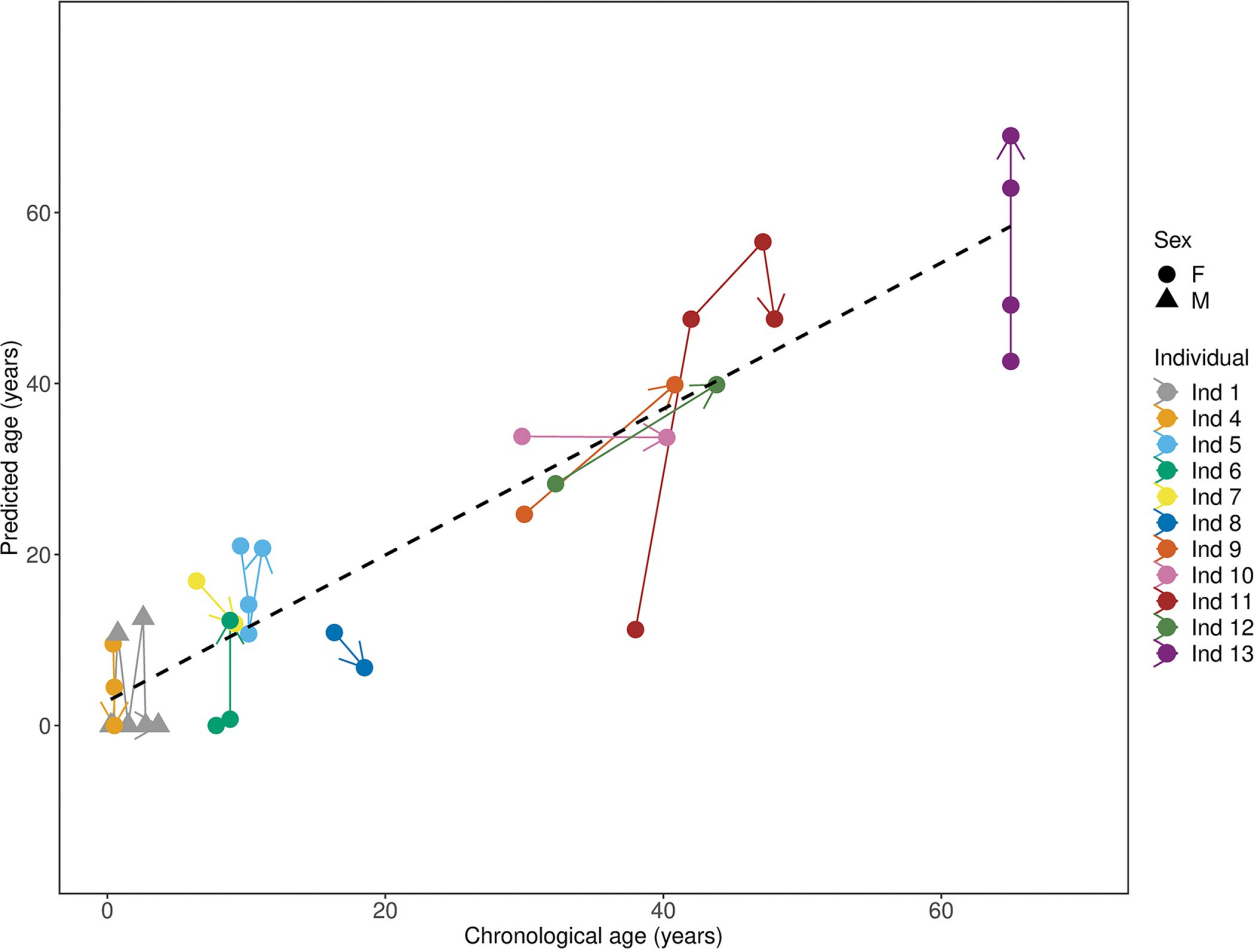
Within-individual changes in DNA methylation with age. Age tracking for the final age estimation model. Predictions for individuals containing at least two blood samples collected over time in the data set (S1 Table). The final model was able to correctly predict 71% (20/28) cases of which samples collected from an individual at a later date were from an older sample. The dashed line represents the overall simple regression analysis between predicted and chronological age. There was a consistent significant relationship between predicted age and chronological age, even with samples collected over time (*R*^2^ = 0.86, *p* <0.001).

We compared the within-individual change from the same individual sampled more than once. Individuals who were sampled multiple times showed similar trends in methylation level changes with age (Fig 7). With a subset of 28 individuals for which two or more samples were collected at different times (S1 Table), the final age estimation model with both *RALYL* and *TET2*, predicted that ages were older for the later-collected samples (*p* = 0.03) (Fig 7), with 71% (20/28) cases that correctly predicted to be from an older aged sample. Moreover, the model, independently and in combination, consistently showed a significant relationship between predicted age and chronological age during within-individual change over time (*R*^2^ = 0.86, *p* <0.001; Fig 7).

## Discussion

Age estimation in animals is important to understand their life-history traits and demographic properties [3,67]. Current methods can be difficult, laborious, subjected to bias, and often only obtainable by field studies over a long period of time [9]. In contrast, current molecular techniques to measure methylation, such as microarrays and RRBS have high costs and require an extended length of time for analysis. This study investigates the relationship between DNA methylation levels of age-related genes and chronological age in Asian elephants using a labour-, time-, and cost-effective molecular technique, namely MS-HRM. One of the unique characteristics of MS-HRM is that it can detect the overall methylation status of the region of interest. Thus, the methylation status of many CpG sites can be detected with a single MS-HRM analysis, while other methylation techniques such as microarrays and pyrosequencing detect only a limited number of CpG sites. By using captive Asian elephants with known ages provided by Japanese zoos, this study further investigates the potential use of MS-HRM in building an age estimation model for Asian elephants. Our results suggest that the two candidate age-related epigenetic genes tested in this study, *RALYL* and *TET2*, showed correlations between chronological age and methylation levels, with the final age estimation model constructed using MS-HRM and LOIOCV being able to estimate age with a MAE value of 7.36 years.

### Age estimation based on two age-responsive genes using MS-HRM

The estimation accuracy and validation of the model improved when the methylation status for both genetic regions, *RALYL* and *TET2*, were combined into the age estimation model instead of separating the regions as independent models (S1 and S2 Files). This was consistent with previous findings where increasing the number of target sites enhanced age prediction accuracy [41,68]. However, the estimation accuracy in this study was lower than in a previous study, which constructed an age estimation model for Asian elephants using a different molecular technique; a DNA microarray chip with a mean absolute error (MAE) of 3.41 years [47]. The relatively low estimation accuracy in this study in comparison to the DNA microarray chip method could be caused by the limited number of genetic regions used (two in comparison to 37 regions). As mentioned previously, MS-HRM has its advantages regarding the time and cost required for the analysis. However, less information is obtained with MS-HRM compared to that in the microarray chip, which measures individual CpG methylation levels within each gene region [41,69]. Knowing the methylation status of each CpG site could be useful, as combinations of CpG sites with the best relationship with age are assumed to increase the accuracy of the age estimation model [70,71]. However, our model has the potential to provide an alternative option to current age estimation methods as we further tested the validation of the final age estimation model (Figs 6 and 7) and revealed that combining both genetic regions could provide a practical value to estimate age using MS-HRM. The preferable number of target sites is suggested to be three for practical reasons [72]. Thus, incorporating at least one more candidate genetic region to establish a more accurate model should be investigated for future applications.

The models tended to overestimate younger individuals and underestimate older individuals (Fig 4). Similarly, previous studies that have attempted to construct an age estimation model using DNA methylation have demonstrated difficulties in accurately predicting chronological age with a small error, especially for older individuals [73,74]. However, other studies have reported little prediction error in younger individuals and observed a similar increase in prediction error as chronological age increases [41]. Change in prediction error across age could be due to younger individuals having a tendency to age at a faster rate. Moreover, samples of older individuals are often difficult to obtain, which could have caused the increase in prediction error in older ages. Although the exact reasons for less precise age prediction among younger and older individuals remain unclear, it is suggested that this might be attributable to individual differences in the rate of methylation change that accumulate with age [41] due to methylation saturation, in which CpG probes reach 0% or 100% methylation later in life, in addition to other age-related processes [74].

In this study, candidate age-related epigenetic genes successfully used in other species were selected to assess methylation rates for Asian elephants. *TET2* is a well-known epigenetic age-related gene that has been used as a region to estimate chronological age across several mammalian species [30,36,37,39]. In the MS-HRM analysis using felids, *RALYL* showed significant correlations with chronological age [28]. Using the same genes, the age estimation model developed in this study showed significant correlations between the methylation rate of each gene and chronological age. However, the specificity may not be as high compared to that in methylation microarrays for the abovementioned reasons and as it quantifies methylation across the genome. This again accounts for the lower estimation accuracy in this study compared to that in the microarray technique. For these reasons, in MS-HRM analysis, careful primer design is required. The primers used in this study were specific to Asian elephants, as they were carefully designed using homogenous gene regions searched against the readily available Asian elephant genome [54]. In addition, *TET2* is an evolutionary conserved gene that belongs to the TET family in mammals [75] and plays a key role in the epigenetic process across several mammals. In contrast, though not well studied, *RALYL* is suggested to be conserved in chimpanzees, rhesus monkeys, dogs, cows, and mice [76]. This suggests that *TET2* and *RALYL* could be conserved in other elephant species, however, primers need to be specific and designed with care when using MS-HRM.

As mentioned in the introduction, MS-HRM is an easy-to-use, traditional polymerase reaction. However, like any other PCR reaction, MS-HRM has the issue of PCR bias, where methylated templates are less effectively amplified than in unmethylated templates. Due to this PCR bias, differences in methylation rates are hard to detect, especially in lower-methylated regions [41]. To avoid this, sequences without any PCR bias need to be identified, or primers that reverse such PCR bias need to be designed. In addition, if CpG sites in a CpG island were intended to be analysed, designing primers might become difficult as they should include as few CpG sites as possible [41]. These issues were observed in the *ZFHX3* gene (S1 Table in S1 File), a candidate gene referred to in the microarray study [47], as the genetic sequence included many CpG sites. This caused difficulties in primer design and could have impacted the bisulphite conversion efficiency. These complications using MS-HRM analysis were observed in most of the genetic regions identified in the microarray study, as it quantified individual CpG sites for each genetic region across the genome.

The use of captive known-age individuals is required to construct the basis of the age estimation model because the equation of methylation status needs to be generated to construct a reliable model. In the case of wild individuals, information on chronological age is expected to be unknown (depending on the area). Then, with the unknown sample, the expected possible deviation range needs to be considered in the equation of the constructed model. An additional inspection to check if the DNA methylation values fall within the expected range would be needed [77]. Studies to test the applicability of the age estimation model have been assessed in certain species and had no significant difference between the methylation rates of captive and wild individuals [78].

### Comparison with other species and other chronological age estimation methods

When we compared the age estimation error (MAD/MAE) to the average life span from previous studies that used MS-HRM to predict age, humans had an accuracy rate of 90.36%, considering its MAD of 7.71 years to an average life span of 80 years [41]. A study using domestic cats showed an accuracy rate of 78.70%, with a MAD of 3.83 years and an average life span of 18 years [28]. In comparison to these previous studies, this study constructed an age estimation model with an accuracy rate of 87.73%, considering the average life span of Asian elephants to be 60 years. When compared to the current methods to estimate age in elephants, dental methods have an accuracy rate of 73.3–90% (error of −6 to +16 years) [13,79] and rumbles with 70% [80]. Age estimation accuracy/error rate of dung measurements was not mentioned. However, it was suggested that due to the high variability in dung measurements in each age class, there were limitations in predicting an age structure for young elephants and those older than 20 years [17]. In addition, the most popular method of estimating elephant age is morphological observations such as shoulder height. However, we could not compare as the accuracy/error rate was not mentioned [9]. Moreover, age estimation by morphological observations could be heavily based on experience. If the observer is inexperienced, the accuracy and precision of the estimated age could be reduced. The only methylation studies on Asian elephants were investigated using methylation microarrays, with a higher accuracy rate of 3.41 years (MAE) compared to our study [47]. However, microarrays require a minimum concentration of 50 ng/μL, with a minimum input of 200–300 ng of bisulphite converted DNA [81]. Considering these requirements, this study only needed 5 ng/μL of bisulphite-converted DNA. Although a DNA input of 300–500 ng was used as a starting material, MS-HRM can measure DNA inputs of 20 ng/gene, while the manufacturer’s protocol for bisulphite conversion states that samples with at least 500 pg could be used. In comparison, another single gene technique similar to MS-HRM; pyrosequencing, requires at least 150 ng of DNA input. However, due to low amplification efficiency, it is recommended that the standard control DNA input should be greater than the sample [46]. With the use of MS-HRM, low DNA input will be beneficial for precious samples, especially for endangered species like Asian elephants. Thus, in comparison to other age estimation methods, MS-HRM has the potential to be used as an alternative technique to estimate age in Asian elephants, considering its advantages of being able to measure age genetically from a wide range of methylation status within the genetic region of interest in a labour-, time-, and cost-effective manner, preventing any observation bias from current age estimation methods.

### Age estimation model limitations

A sample size of at least 70 or more is suggested [82], as it could decrease the MAE, standard deviation, and relative error, leading to improved accuracy. The small sample size reflects the difficulty of obtaining samples across Japan due to the limited number of individuals in captivity. Furthermore, husbandry and management should be considered within zoos across Japan, as mentioned in the methods, along with the longevity and life-history status of the elephants. Due to the limited number of individuals, our model lacked equal representation between age groups, especially between 15–30 and 40–50 years. However, one of the advantages of this study was the comparison of individual changes across individuals sampled multiple times over the years, with the final age estimation model showing increasing methylation levels with increasing age within the same sampled individual.

In this study, sex did not affect the final age estimation model. This finding indicates that no sex difference existed in age-dependent DNA methylation changes or individual variability. Previous studies have contradicting results on sex-specific age-related DNA methylation changes. Among humans, a few studies have indicated sex-specific age-related DNA methylation changes [83], while others indicate no significant difference [43]. Several studies have suggested that model accuracy improved when separating males and females in a study from felines [28], while others have suggested that separating sex does not significantly improve the age estimation model [36,78]. Notably, this study had small sample sizes for males (*n* = 10) compared to females (*n* = 43) which could have considerably impacted whether the model was indeed age-dependent or not. The small sample size was reflected by the small captive male populations of approximately only 20 individuals across Japan, which hinders sample collection. Further validation of the age estimation model on an independent data set with a balanced sex ratio is required before establishing this method as a widespread tool. Increasing samples from more known-age individuals, especially in the age range between 15–30 and 40–50 years, and increased representation of sex could foster further development and refinement of the model. However, it should be noted that other age estimation models with low sample sizes (<70 samples) still produced an age estimation model with small errors [30,33,37,84]. Furthermore, highly accurate or precise models may not be required for all applications [6,33], as it ultimately comes to how the model is being used and for what purpose.

### Future perspectives

Approximately 82% of the variation observed in DNA methylation patterns among Asian elephants in the constructed model could be explained by the difference in chronological age. The remainder of the variation (18%) could potentially be due to other factors, such as stress exposures in captivity and/or genetic variability. Recent findings suggest that DNA methylation of certain genetic regions can be influenced by biological, social, and environmental factors [85]. In contrast, others are linked to genetically programmed ageing and are less influenced by such factors. For example, multiple studies have shown that DNA methylation pattern can be altered by disease status (e.g., cancer, cardiovascular disease, diabetes, and Alzheimer’s disease), demographic and lifestyle characteristics (e.g., race, smoking, and alcohol consumption), diet, and environmental exposures (e.g., sun exposure and air pollution) in humans [85–87]. In non-human animals, these aspects are not as well investigated. However, there are studies on the effects of social status in naked mole rats (*Heterocephalus glaber*) and baboons (*Papio*) [88,89], hibernation in ground squirrels (*Ictidomys tridecemlineatus*), brown bats (*Eptesicus fuscus*), and yellow-bellied marmots (*Marmota flaviventer*) [90–92], and temperature change in zebra finches (*Taeniopygia guttata*) [93] on DNA methylation patterns. Thus, different tendencies in DNA methylation patterns between individuals could be affected by different factors. However, in this study, other than sex, other biological, social, or environmental factors were not considered. To assess the methylation of certain genes as a useful marker, further studies must evaluate and examine the differences between chronological age and biological age, and the factors that cause them.

Epigenetic age estimation models have been well investigated using blood and tissue samples. High age correlation has consistently been observed in heterogeneous tissues (such as blood, brain, buccal epithelium, saliva, liver, lung, and kidney), with blood, brain, and saliva samples demonstrating slightly higher accuracy [18,94]. However, most of these studies are based on methylation arrays, and using brain, liver, lung, or kidney samples is not idealistic to estimate age for future conservation purposes. To our knowledge, only studies on blood and saliva samples use MS-HRM to estimate age [28,41,46]. In Hamano et al., the model that used saliva samples demonstrated a slightly higher accuracy with a difference of 1.46 years (MAD) than in the model developed with blood samples [41,46]. However, we assume this is most likely due to the larger sample size. As our model was constructed using blood samples, the accuracy might not significantly differ from other tissues for the abovementioned reasons unless the sample size and number of genetic regions increase. However, using the age estimation model for conservation efforts constructed in this study is currently limited as it can only be applied to blood samples. DNA methylation is suggested to be chemically stable compared to other age-related biomarkers as it has been assayed from historic bone samples or ancient DNA from 38,000–60,000 years ago [95,96]. This could suggest that methylation biomarkers may be more suitable for age estimation tools in comparison to other age estimation techniques. MS-HRM studies using faecal samples have only been performed in humans [97]. No studies have been made to assess the use of faecal samples to estimate age through methylation data. Although model accuracy may not be as high as in heterogeneous tissues due to DNA quality, developing a non-invasive molecular technique to estimate age from degraded DNA, such as faecal samples would still be valuable. Extending age estimation techniques to non-invasive samples, such as faecal samples would enable researchers to collect samples in the field without disturbing the animals, avoid long-term visual surveys, and limit distress to a minimum. When developing an age estimation model from different types of samples, it is advised not to apply an age estimation model developed, for example, saliva samples to blood samples or mixed samples as methylation profiles can change [46]. Nevertheless, it would still be worthwhile to explore the development of age estimation models using DNA methylation from low or non-invasive samples, such as hair or faecal samples using MS-HRM for future use in conservation efforts.

## Conclusions

The objective of this study was to assess the ability of MS-HRM as a method to determine DNA methylation levels and estimate chronological age in Asian elephants. Though exploratory, this study demonstrates the possibility of assessing chronological age using MS-HRM as an alternative age estimation method for Asian elephants. The age estimation model constructed with MS-HRM using LOIOCV showed correlations between chronological age and methylation levels and obtained a MAE value of 7.36 years. This study opens an avenue to further explore the use of MS-HRM to estimate age, as a certain level of prediction can be performed with comparable accuracy rates to models established for other mammals using MS-HRM and observational age estimation methods used for Asian elephants. MS-HRM has its advantages, as it is easy to use and requires less time and cost for methylation analysis. However, less information is obtained with MS-HRM compared to that in other molecular techniques measuring CpG methylation rates, which was evident from the lower estimation accuracy compared to the microarray study. Nevertheless, our model has the potential to provide another alternative for predicting chronological age. These findings and interpretations could be further improved by enhancing the sampling effort (adding more individuals/samples, equal representation of sex and age groups), increasing the number of candidate age-related genes, and integrating predictor variables to account for potential variability. Further age estimation model development using non-invasive samples could illuminate possibilities for applications to wildlife populations and may supplement conservation implications. Further exploration of MS-HRM as an age estimation technique could facilitate advancements over alternatives for Asian elephants and other species.

## Supporting information

S1 Dataset Raw data set of methylation rate. (XLSX)

S1 File List of supplementary tables. (DOCX)

S2 File List of supplementary figures. (DOCX)

S1 Table Details on samples and individuals. Summary of blood sample information (*n* = 53) taken from the studied captive Asian elephants (*n* = 25) between 2004 and 2022 from Japanese zoos.
(DOCX)
