# Supplementary material for: Age estimation of captive Asian elephants (Elephas maximus) based on DNA methylation: An exploratory analysis using methylation-sensitive high-resolution melting (MS-HRM)
Source: PLoS One. 2023 Dec 11;18(12):e0294994. doi: 10.1371/journal.pone.0294994 (PMC10712859; doi:10.1371/journal.pone.0294994)
Supplement: S1 File — (DOCX) [file pone.0294994.s002.docx]

**S1 Table. Primer information.** Additional primer information was tested using other genes; however, results from the real-time polymerase chain reaction (PCR) were unsatisfactory.

| **Gene** | **Primers**^a^ | **Length** | **n(CpGs)** | **NCBI sequence ID: position** | **References** |
| --- | --- | --- | --- | --- | --- |
| *TET2* | F: ggaagggTgTagTTagagagTTTagaa  R: tgtggagaggttattgagaagaaag  R: gggtatttggatttatagggagtg | 218  283 | 4  11 | NC_064833.1: 46696760–46696879 | Polanowski et al., (2014); Wright et al., (2018) |
| *ELOVL2* | F: ggCgtttggTTagTagggT  R: tggtcttggtggttttcaaaca  R: tggtcttggtggttttcaaaca | 204  208 | 14  29 | NC_064833.1: 54556091–54556299 | Bekaert et al., (2015); Qi et al., (2021a) |
| ZFHX3 | F: ctggcagctggggctacTtt  R: tgtgcttgcaaaggatggag  F: gggtagagTagTagTTTatTtggag  R: agagctgtgtgtgggacctgag  F: gagagTtgtgtgtgggaTTtgag  R: gggaaggacaatgggtgC  R: gggcccctggacagcctgaggg | 139  238  192  311 | 4  6  7  11 | NC_064833.1:  37563009–37563698 | Prado et al., (2021) |

^a^Capital letter: bisulfite-converted letter (F: c → T, R: g → A)

**S2 Table. Optimised parameters of support vector regression (SVR) models.**

|  | **Cost** | **Gamma** | **Epsilon** |
| --- | --- | --- | --- |
| **Final age estimation model (*RALYL* + *TET2*)** | 316.23 | 0.5 | 0.2 |
| ***RALYL* model** | 100 | 1 | 0.5 |
| ***TET2* model** | 158.49 | 1 | 0.5 |

**S3 Table. Details of standard curves.** Values of the coefficient ‘a’ in Equation 1, the *R*^2^, and *p*-values of the standard curve for each gene region.

|  | a | *R*^2^ | *p*-value |
| --- | --- | --- | --- |
| *RALYL* | 0.59 | 0.99 | **<0.001** |
| *TET2* | 0.47 | 0.95 | **<0.001** |

**S4 Table. Linear regression output for each age estimation model.**

| **Model** | **Variable** | **Estimated coefficients** | **Standard error** | **95% CI** | **R^2^_adjusted_** | ***p* - value** |
| --- | --- | --- | --- | --- | --- | --- |
| *RALYL* | (Intercept) | 1.917 | 5.817 |  |  |  |
|  | LOIOCV_RALYL | 0.876 | 0.225 | 0.425, 1.327 | 0.215 | **<0.001** |
| *TET2* | (Intercept) | 6.271 | 4.587 |  |  |  |
|  | LOIOCV_TET2 | 0.699 | 0.168 | 0.361, 1.037 | 0.238 | **<0.001** |
| *RALYL* + *TET2* (SVM model) | (Intercept) | 2.084 | 1.756 |  |  |  |
|  | all_trial | 0.964 | 0.062 | 0.839, 1.089 | 0.821 | **<0.001** |
| *RALYL* + *TET2* (after LOIOCV) | (Intercept) | 0.733 | 2.279 |  |  |  |
|  | LOIOCV_all_trial | 0.978 | 0.081 | 0.816, 1.140 | 0.737 | **<0.001** |

**S5 Table. Linear regression output for each age estimation model on the influence of sex and chronological age on each model.**

| **Model** | **Variable** | **Estimated coefficients** | **Standard error** | **95% CI** | ***p* - value** |
| --- | --- | --- | --- | --- | --- |
| *RALYL* | Δage residual |  |  |  |  |
|  | (Intercept) | -1.503 | 2.518 |  |  |
|  | Age | 0.035 | 0.035 | -0.118, 0.187 | 0.647 |
|  | Sex | 3.792 | 3.792 | -3.877, 11.460 | 0.325 |
| *TET2* | Δ age residual |  |  |  |  |
|  | (Intercept) | 0.774 | 3.287 |  |  |
|  | Age | -0.018 | 0.099 | -0.217, 0.181 | 0.857 |
|  | Sex | -1.953 | 4.984 | -11.964, 8.058 | 0.697 |
| *RALYL* + *TET2* | Δage |  |  |  |  |
|  | (Intercept) | -0.014 | 2.367 |  |  |
|  | Age | 3.295 × 10^-4^ | 0.071 | -0.143, 0.144 | 0.996 |
|  | Sex | 0.036 | 3.590 | -7.175, 7.246 | 0.992 |

**S6 Table. Linear regression output for each within-individual change assessment.**

| **Model** | **Variable** | **Estimated coefficients** | **Standard error** | **95% CI** | ***R*^2^_adjusted_** | ***p* - value** |
| --- | --- | --- | --- | --- | --- | --- |
| *RALYL* | (Intercept) | -2.379 | 5.599 |  |  |  |
|  | Individual_RALYL | 1.056 | 0.221 | 0.611, 1.501 | 0.308 | **<0.001** |
| *TET2* | (Intercept) | 1.859 | 4.617 |  |  |  |
|  | Individual_TET2 | 0.812 | 0.161 | 0.489, 1.135 | 0.334 | **<0.001** |
| *RALYL + TET2* | (Intercept) | 0.081 | 1.710 |  |  |  |
|  | Individual_both | 1.007 | 0.058 | 0.890, 1.125 | 0.858 | **<0.001** |
