# Supplementary material for: Age estimation of captive Asian elephants (Elephas maximus) based on DNA methylation: An exploratory analysis using methylation-sensitive high-resolution melting (MS-HRM)
Source: PLoS One. 2023 Dec 11;18(12):e0294994. doi: 10.1371/journal.pone.0294994 (PMC10712859; doi:10.1371/journal.pone.0294994)
Supplement: S2 File — (DOCX) [file pone.0294994.s003.docx]

**
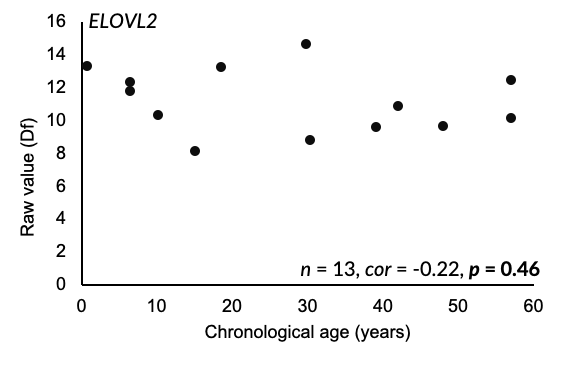
**

**S1 Fig. Unsuccessful example after methylation-sensitive high-resolution melting (MS-HRM) trial runs using *ELOVL2*.** DNA samples (*n* = 13) isolated from blood were analysed as a trial run; however, there was no correlation between Df values and chronological age.

**S2 Fig. Age estimation model for each genetic region.** Relationship between predicted age and chronological age for each genetic region after leave-one-individual-out cross-validation (LOIOCV) analysis for (A) *RALYL* and (B) *TET2*. The black line represents the y = x diagonal line. The region between the grey dashed lines is the mean absolute error (MAE) range: (A) 12.39 years (*R*^2^ = 0.22, *p* <0.001), and (B) 8.92 years (*R*^2^ = 0.24, *p* <0.001).

**S3 Fig. Influence of sex on each single gene model.** Δage residuals, defined as the residual from regressing predicted age on chronological age was calculated. Chronological age was adjusted as a covariate. Figures on the left compare the distribution of Δage residuals between females and males in (A) *RALYL* and (C) *TET2*. The boxplots show group medians (solid line), inter-quartile range (box outline) and spread of data with outliers (whiskers) for each group. Figures on the right show the relationship between Δage residuals and chronological age in (B) *RALYL* and (D) *TET2*. After linear regression analysis, sex (*RALYL*: *p* = 0.325; *TET2*: *p* = 0.697) and chronological age (*RALYL*: *p* = 0.647; *TET2*: p = 0.857) adjusted as a covariate did not affect Δage residuals significantly.

**S4 Fig. Box plots representing the predicted age of Asian elephants categorised into four age classes in each single gene age estimation model.** Box plots represent the predicted age of captive Asian elephants categorised into four categories of known age: calf (<1 year), juvenile (1–5 years), subadult (5–15 years), and adult (>15 years). The boxplots show group medians (solid line), inter-quartile range (box outline) and spread of data with outliers (whiskers) for each group. (A) *RALYL* model had moderate predictive power with a kappa value of 0.437 (0.231–0.645, *p* <0.001). The model could differentiate age classes (ANOVA: *F* = 3.34, *p* <0.01), specifically between subadult and adult (**p* <0.05). (B) *TET2* model showed poor predictive power with a kappa value of 0.191 (-0.023–0.404, *p* = 0.08) and was unable to differentiate between age classes.

**S5 Fig. Within-individual changes in DNA methylation with age in each single gene model.** Age tracking for the single age estimation models. Predictions for individuals containing at least two blood samples collected over time in the data set (S1 Table). For both single gene models, the predicted ages were not older for the later collected samples with (A) *RALYL* predicting only 57 % (16/28) cases (*p* = 0.57) and (B) *TET2* with 46 % (13/28) cases (*p* = 0.85) of which samples collected from an individual at a later date were from an older sample. The dashed line represents the overall simple regression analysis between predicted and chronological age. Both single gene models, independently and in combination, consistently showed a significant relationship between predicted age and chronological age during within-individual change over time (*RALYL*: *R*^2^ = 0.308, *p* <0.001; *TET2*: *R*^2^ = 0.334, *p* <0.001).
