# Supplementary material for: Age estimation of captive Asian elephants (Elephas maximus) based on DNA methylation: An exploratory analysis using methylation-sensitive high-resolution melting (MS-HRM)
Source: PLoS One. 2023 Dec 11;18(12):e0294994. doi: 10.1371/journal.pone.0294994 (PMC10712859; doi:10.1371/journal.pone.0294994)
Supplement: S1 Table — Summary of blood sample information (n = 53) taken from the studied captive Asian elephants (n = 25) between 2004 and 2022 from Japanese zoos. (DOCX) [file pone.0294994.s004.docx]

**S1 Table. Details on samples and individuals.**

Summary of blood sample information (*n* = 53) taken from the studied captive Asian elephants (*n* = 25) between 2004 and 2022 from Japanese zoos.

| Individual | Sex | Sampling date | Chronological age (years) | Predicted age  (years)^a^ | Major age class^b^ |
| --- | --- | --- | --- | --- | --- |
| 1 | M | 2008/01/20 | 0.25 | 0 | Calf |
|  |  | 2008/01/31 | 0.25 | 0 | Calf |
|  |  | 2008/03/02 | 0.417 | 0 | Calf |
|  |  | 2008/07/23 | 0.75 | 10.72 | Calf |
|  |  | 2009/03/16 | 1.5 | 0 | Juvenile |
|  |  | 2010/02/04 | 2.583 | 12.56 | Juvenile |
|  |  | 2010/05/26 | 2.75 | 0 | Juvenile |
|  |  | 2011/06/13 | 3.67 | 0 | Juvenile |
| 2 | M | 2018/05/28 | 6.583 | 13.40 | Sub-adult |
| 3 | M | 2022/01/01 | 17.583 | 25.41 | Adult |
| 4 | F | 2004/08/26 | 0.417 | 9.54 | Calf |
|  |  | 2004/09/04 | 0.5 | 0 | Calf |
|  |  | 2004/09/21 | 0.5 | 4.47 | Calf |
| 5 | F | 2017/10/21 | 9.583 | 21 | Sub-adult |
|  |  | 2018/05/07 | 10.167 | 10.73 | Sub-adult |
|  |  | 2018/05/09 | 10.167 | 14.13 | Sub-adult |
|  |  | 2018/05/20 | 10.167 | 14.15 | Sub-adult |
|  |  | 2019/05/19 | 11.167 | 20.74 | Sub-adult |
| 6 | F | 2018/05/24 | 7.83 | 0 | Sub-adult |
|  |  | 2019/05/19 | 8.83 | 0.74 | Sub-adult |
|  |  | 2019/05/20 | 8.83 | 12.29 | Sub-adult |
| 7 | F | 2016/08/30 | 6.417 | 11.92 | Sub-adult |
|  |  | 2019/05/14 | 9.167 | 16.90 | Sub-adult |
| 8 | F | 2006/08/27 | 16.33 | 10.88 | Adult |
|  |  | 2008/10/26 | 18.5 | 6.77 | Adult |
| 9 | F | 2010 | 30 | 24.70 | Adult |
|  |  | 2021/11/07 | 40.83 | 39.85 | Adult |
| 10 | F | 2010 | 29.83 | 33.81 | Adult |
|  |  | 2022/01/27 | 40.25 | 33.69 | Adult |
| 11 | F | 2009/05/08 | 38 | 11.22 | Adult |
|  |  | 2013/10/28 | 42 | 47.53 | Adult |
|  |  | 2018/05/09 | 47.167 | 56.56 | Adult |
|  |  | 2019/05/14 | 48 | 37.48 | Adult |
|  |  | 2019/10/27 | 48 | 47.54 | Adult |
| 12 | F | 2009 | 32.25 | 28.27 | Adult |
|  |  | 2009 | 32.25 | 36.23 | Adult |
|  |  | 2022/03/12 | 43.83 | 39.86 | Adult |

**S1 Table. (continued)**

| 13 | F | 2008/04/04 | 65 | 42.58 | Adult |
| --- | --- | --- | --- | --- | --- |
|  |  | 2008/04/07 | 65 | 49.18 | Adult |
|  |  | 2008/04/08 | 65 | 62.85 | Adult |
|  |  | 2008/04/09 | 65 | 68.97 | Adult |
| 14 | F | 2021/12/02 | 6.417 | 14.16 | Sub-adult |
| 15 | F | 2022/01/10 | 8.917 | 22.62 | Sub-adult |
| 16 | F | 2021/12/02 | 10.75 | 16.78 | Sub-adult |
| 17 | F | 2009/06/25 | 11 | 25.76 | Sub-adult |
| 18 | F | 2009/06/24 | 15 | 18.93 | Sub-adult |
| 19 | F | 2022/01/07 | 20.167 | 16.19 | Adult |
| 20 | F | 2021/12/09 | 30.33 | 43.71 | Adult |
| 21 | F | 2009/06/29 | 32 | 14.42 | Adult |
| 22 | F | 2023/03 | 38 | 29.50 | Adult |
| 23 | F | 2009/07/17 | 39 | 9.01 | Adult |
| 24 | F | 2010/06/07 | 42 | 36.65 | Adult |
| 25 | F | 2021/12/09 | 44 | 39.98 | Adult |

^a^ Predicted age taken from the final age estimation model (including both genes).

^b^ Categorizing Asian elephant individual into respective age class was referred to Arivazhagan, C., and Sukumar, R. (2008).
